# Pose-Based Static Sign Language Recognition with Deep Learning for Turkish, Arabic, and American Sign Languages

**DOI:** 10.3390/s26020524

**Published:** 2026-01-13

**Authors:** Rıdvan Yayla, Hakan Üçgün, Mahmud Abbas

**Affiliations:** Bilecik Şeyh Edebali University, Faculty of Engineering, Department of Computer Engineering, Bilecik 11100, Türkiye; hakan.ucgun@bilecik.edu.tr (H.Ü.); mahmodabas070@gmail.com (M.A.)

**Keywords:** sign language recognition, human-computer interaction, pattern recognition, applications of gesture recognition, hand gesture recognition, MediaPipe

## Abstract

Advancements in artificial intelligence have significantly enhanced communication for individuals with hearing impairments. This study presents a robust cross-lingual Sign Language Recognition (SLR) framework for Turkish, American English, and Arabic sign languages. The system utilizes the lightweight MediaPipe library for efficient hand landmark extraction, ensuring stable and consistent feature representation across diverse linguistic contexts. Datasets were meticulously constructed from nine public-domain sources (four Arabic, three American, and two Turkish). The final training data comprises curated image datasets, with frames for each language carefully selected from varying angles and distances to ensure high diversity. A comprehensive comparative evaluation was conducted across three state-of-the-art deep learning architectures—ConvNeXt (CNN-based), Swin Transformer (ViT-based), and Vision Mamba (SSM-based)—all applied to identical feature sets. The evaluation demonstrates the superior performance of contemporary vision Transformers and state space models in capturing subtle spatial cues across diverse sign languages. Our approach provides a comparative analysis of model generalization capabilities across three distinct sign languages, offering valuable insights for model selection in pose-based SLR systems.

## 1. Introduction

Sign language (SL) has been a fundamental and highly expressive form of communication for individuals with hearing challenges. According to the World Health Organization (WHO) (Geneva, Switzerland), over 432 million adults and 34 million children—more than 5% of the global population—experience some form of hearing difficulty [1]. This number is projected to exceed 700 million by 2050 [2], underscoring the growing need for inclusive and assistive communication technologies.

Recent advances in artificial intelligence (AI) have paved the way for developing intelligent systems that enhance accessibility and communication. AI-driven applications, particularly in the fields of computer vision and machine learning, have shown significant promise in supporting individuals with hearing-related challenges. Sign language recognition (SLR) systems are among these promising technologies, offering the potential for real-time translation of gestures into textual or spoken language to bridge communication gaps.

While the field has rapidly advanced toward continuous, video-based sign language translation (SLT), robust recognition of static hand poses has remained a fundamental and challenging task. It is important to clarify that this study focuses exclusively on isolated fingerspelling and manual sign recognition, where each sign has been defined by a distinct, static hand configuration, and temporal dynamics have not been the primary distinguishing factor. This has been particularly true for practical and real-time applications such as digital communication assistance and educational tools focusing on finger spelling and isolated manual signs. Although modern SLR often depends on movement and dynamics (coarticulation), our study has focused specifically on Pose-Based Static SLR using still frames to establish a lightweight, low-latency foundation crucial for embedded and edge-device real-time alphabet recognition. Consequently, dynamics and co-articulation, while critical for full SLR, have been outside the scope of this isolated sign recognition benchmark.

This study has focused on the design and evaluation of a framework that utilizes MediaPipe-based landmark detection in combination with cutting-edge deep learning (DL) models. Our approach has provided a cross-lingual analysis by training independent models for three distinct linguistic groups: Turkish Sign Language (TSL), Arabic Sign Language (ArSL), and American Sign Language (ASL). In contrast to multilingual training systems that require joint training of representations and parameter sharing, our work has critically assessed the generalization capabilities and architectural biases of modern state-of-the-art architectures—specifically ConvNeXt, Swin Transformer, and Vision Mamba (Vmamba)—when applied to identical, pose-based feature sets derived from significantly different sign languages.

### 1.1. Novelty of the Research

While previous studies have extensively analyzed sign language recognition using various algorithms, a literature gap has remained regarding integrated, cross-lingual frameworks that adaptively support distinct languages such as Arabic, Turkish, and American sign languages. Most existing research has relied on single-source datasets and has lacked comparative evaluations using state-of-the-art architectures. To address this challenge, this study has introduced a unified platform powered by the Vision Mamba (Vmamba) architecture, demonstrating superior performance and addressing the critical need for robust, cross-lingual sign language recognition systems.

This study has provided significant contributions to the field of sign language recognition, both in terms of architectural approach and scope.

1. First, one of the first integrated systems in the literature has been developed, dynamically supporting Arabic (ArSL), Turkish (TSL), and American (ASL) sign languages under a single, unified interface and architectural framework. This approach has demonstrated the capability to offer an adaptive solution for sign language users across diverse geographical and cultural regions.
2. Second, enhanced language-specific datasets have been established, meticulously collected and labeled from various sources for each language (Arabic, Turkish, English). These datasets constitute a major academic foundation and serve as a valuable resource for future language-specific sign language research.
3. Finally, by conducting separate training processes for each language, state-of-the-art visual recognition architectures—specifically ConvNeXt, Swin Transformer, and the novel Vision Mamba—have been meticulously compared against strong deep learning baselines. The superior performance and high-performance potential of Vision Mamba in this domain have been comprehensively demonstrated.

Crucially, the primary contribution of this work has been the establishment of Vision Mamba (ViM) as a new, highly effective benchmark for static pose-based Sign Language Recognition (SLR). While ViM’s core architecture has remained unmodified, our study has been the first to rigorously demonstrate its superior cross-lingual generalization capability against established vision models (ConvNeXt and Swin) across three distinct language families (Turkish, Arabic, and American Sign Language). The robustness of ViM in extracting features from sparse 2D pose data, which indicates a new direction for lightweight, high-accuracy static SLR systems, has represented the core novelty of this experimental evaluation.

### 1.2. Contribution

The main contributions of this study have been summarized as follows:

- The construction of a high-quality, diverse dataset for pose-based static SLR spanning TSL, ArSL, and ASL, compiled from nine public domain sources and carefully curated to ensure data diversity.
- A comprehensive cross-lingual comparative analysis of the performance of modern vision architectures—including ConvNeXt, Swin Transformer, and the novel Vision Mamba (Vmamba)—applied to identical MediaPipe-extracted features.
- The demonstration of Vmamba’s superior performance, establishing new state-of-the-art benchmark results for TSL and ArSL static sign language recognition, thereby showcasing the effectiveness of structured state space models for hand pose feature classification.
- An analysis of the trade-offs associated with utilizing static frames for SLR compared to sequence-based methods, supporting the work’s focus on achieving a lightweight, low-latency foundation suitable for real-time finger spelling and resource-constrained environments. This includes a preliminary quantitative evaluation of the models’ throughput and memory usage characteristics.

### 1.3. Literature Review

Sign language (SL) has been an active research domain spanning multiple disciplines, including computer science. The literature has presented a diverse range of studies on SL systems. Pal and Kakade developed a real-time dynamic hand gesture detection system incorporating Kinect sensors, OpenCV, Arduino, and DC servo motor actions [3]. Indriani et al. suggested utilizing MediaPipe for hand gesture recognition to enhance user guide application usability and transform manual user guide applications into more interactive ones [4].

Li et al. presented a single network model called Pose-Anchor that makes use of the New Zealand Sign Language Dataset (NZSL) and the large-scale multiview 3D hand pose dataset (LSM-HPD). This system has detected hand keypoints using a fully convolutional neural network and a region proposal network (RPN) within a Faster R-CNN framework [5]. Martinez and Espejo demonstrated that convolutional neural networks (CNNs) outperformed recurrent neural networks (RNNs) in Spanish Sign Language recognition, achieving 96.42% accuracy [6].

Das et al. presented a static solution for detection of American Sign Language (ASL) using a convolutional neural network trained on 1815 images representing 26 English alphabets [7]. Raheja et al. developed a real-time dynamic Indian Sign Language recognition system utilizing depth information, image segmentation, and a support vector machine (SVM) algorithm [8].

A system for detection of hand gestures in American Sign Language (ASL) was created by Sundar and Bagyammal using MediaPipe and LSTM networks [9]. Huu et al. proposed a system that recognizes ASL letters and converts them from text to speech using LSTM and MediaPipe [10]. Bora et al. proposed a method for detection of gestures in Assamese Sign Language, one of India’s 22 officially recognized languages, using MediaPipe and a feedforward neural network [11]. Rodriguez et al. created a dataset for the first ten numerical digits and 29 different characters in Mexican Sign Language (MSL). Additionally, they applied ML and DL techniques for recognizing MSL signs based on MediaPipe [12]. Luqman introduced an encoder-decoder model called ArabSign for Arabic Sign Language detection using a Kinect V2 camera [13]. The AUTSL study significantly contributed a large-scale, word-level Turkish Sign Language dataset, which has been advantageous for high-granularity sign recognition; however, its reliance on the Kinect v2 sensor for data collection has limited its direct applicability to markerless, RGB-only modern systems [14]. Liu et al. suggested using a two-stream network and keyframe extraction techniques to recognize sign language [15]. Priya and Sandesh developed a sign language word recognition system employing a Support Vector Machine (SVM) classifier with hand images captured by a web camera [16]. Alyami et al. conducted a comprehensive analysis of 25 years of research on Continuous Sign Language Recognition (CSLR), highlighting advancements, challenges, and future opportunities [17].

On the other hand, recent studies focusing on TSL, particularly those leveraging the AUTSL corpus, have demonstrated exceptional performance; for instance, a study achieving 98.6% accuracy on word-level TSL recognition [18,19], underscoring TSL’s compatibility with advanced deep learning methodologies. It has been crucial to note that these high-accuracy results, such as the Logos approach [20] and the TSL studies [18,19], have primarily focused on video-based, continuous, or word-level dynamic sign language recognition. In contrast, this study has been deliberately constrained to static pose-based recognition of finger-spelling elements, prioritizing low-latency, real-time applicability, and simplicity over the complexities of full temporal modeling required for dynamic signs, thereby addressing a different application domain (real-time finger-spelling) within the broader SLR field.

Furthermore, large-scale studies such as the one focusing on the WLASL corpus, which utilized a pre-trained approach in the Logos framework, have demonstrated a significant benchmark accuracy of 66% on this challenging, diverse dataset [20].

As seen in Table 1, the current literature on sign language recognition has often been constrained by limitations in modeling temporal dynamics, handling complex finger spelling, ensuring real-time performance with efficient architectures, and avoiding dependency on specialized hardware. To address these identified gaps, a novel and comprehensive framework has been developed in this study. The proposed approach has leveraged a unified architecture capable of concurrently processing both spatial and temporal features, which has effectively captured the continuous and dynamic nature of signs. Furthermore, the model has been designed to be end-to-end trainable, eliminating the computational overhead associated with multi-stage pipelines and enhancing suitability for real-time application. By utilizing only standard RGB camera input, the system has been optimized for high accessibility and cost-effectiveness, offering a robust and advanced solution that overcomes the critical limitations observed in prior works.

The literature has demonstrated a broad array of SL systems, each primarily focusing on a single sign language. In contrast, this study has aimed to develop a multilingual SL recognition system that integrates Turkish, American, and Arabic sign languages, targeting individuals with hearing impairments who are bilingual or multilingual. The system’s performance has been evaluated using various deep learning methods (especially Vision Mamba) to enhance the detection accuracy of letter signs.

The remainder of this study has been organized as follows: Section 2 outlines the materials and methods, while Section 2.6 provides a detailed explanation of system design and components. Section 3 presents experimental results and finally Section 4 contains a discussion.

## 2. Materials and Methods

This study has introduced a robust framework for Pose-Based Static Sign Language Recognition (SLR) focusing on three distinct languages: Turkish Sign Language (TSL), Arabic Sign Language (ArSL), and American Sign Language (ASL). The core methodology has been centered on a cross-lingual comparative analysis of state-of-the-art deep learning (DL) architectures: ConvNeXt, Swin Transformer, and the novel Vision Mamba (Vmamba). Crucially, this work has involved training three independent models, one dedicated to the static sign alphabet of each language. This approach has been intentionally chosen over joint multilingual training to ensure a clean, isolated assessment of architectural performance. By preventing the feature leakage that complex, shared representation spaces might introduce, we have been able to accurately compare model efficiency and generalization capacity against the specific pose variations inherent to each linguistic dataset.

The strategic choice to focus exclusively on static images (still frames) has been foundational for this research, aiming to establish a benchmark for low-latency, real-time finger spelling recognition. This approach has minimized the computational demand associated with sequential (video-based) processing, making the resultant system highly suitable for implementation on resource-constrained platforms such as embedded devices and mobile applications. The recognition pipeline has been divided into two main stages: (1) robust hand landmark extraction using the MediaPipe framework, and (2) classification of the resulting normalized 2D pose features using the selected DL models.

**Figure 1:**
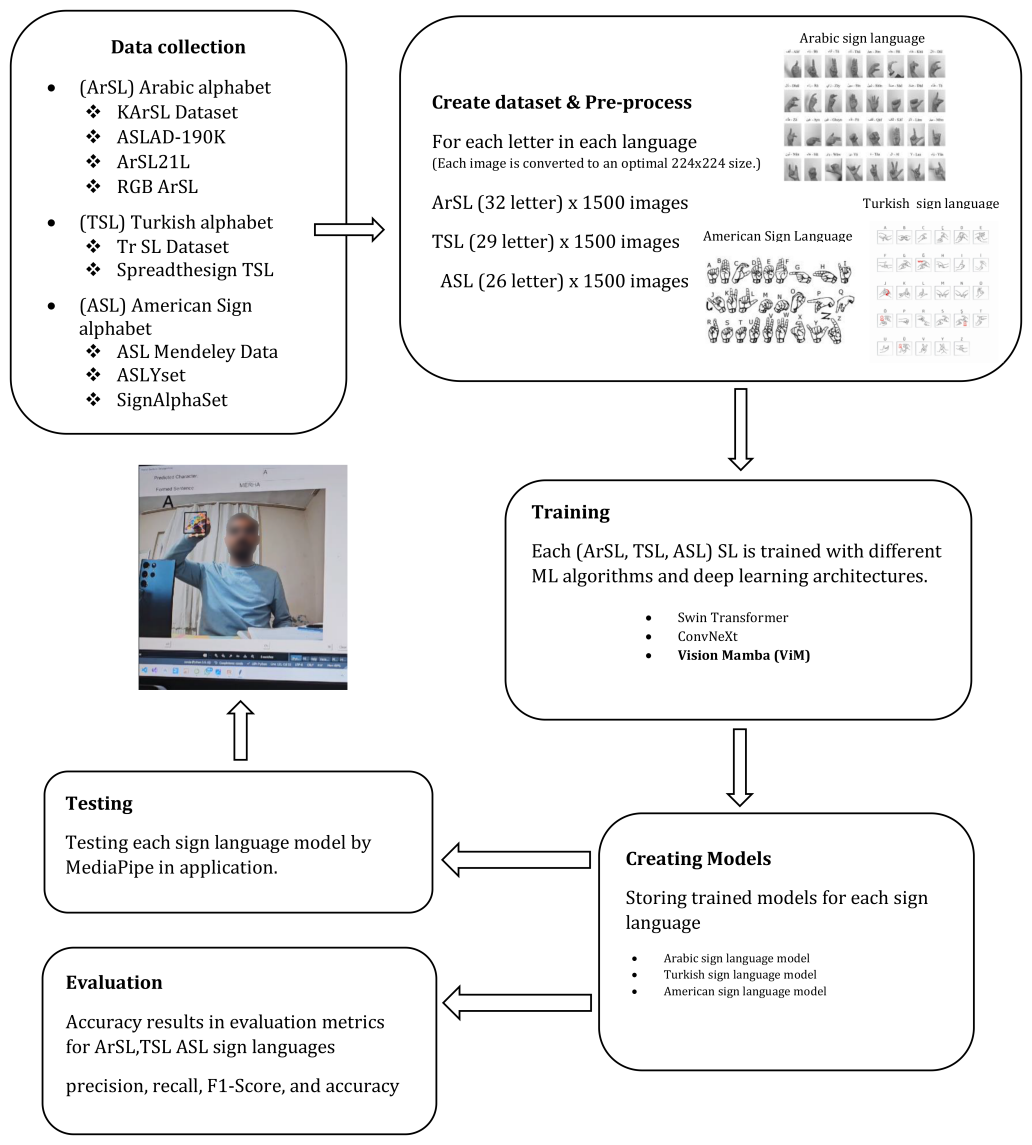
Working principle of sign language recognition.

In this paper, the performance of the three cutting-edge DL architectures has been meticulously compared across the TSL, ArSL, and ASL datasets. This comparative study has aimed to identify the most efficient and accurate model for static pose-based SLR. The system’s operational flow, from image capture to final prediction, has been conceptually outlined in Figure 1.

### 2.1. MediaPipe: Hand Landmark Extraction Layer

**Figure 2:**
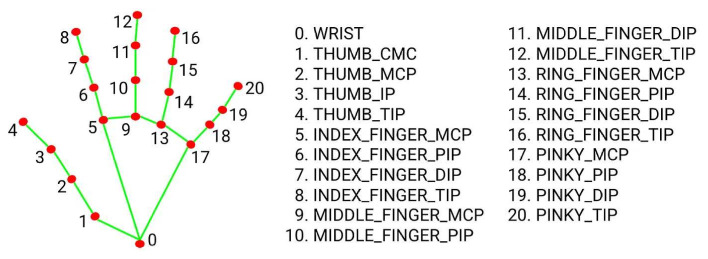
MediaPipe 21 hand landmarks diagram [30]. The landmarks provide the normalized 2D coordinates (x, y) of the hand joints, serving as the input features for the classification models.

MediaPipe has been a robust, open-source framework developed by Google, renowned for its efficient, cross-platform solutions for perception tasks. Specifically, the MediaPipe Hands component has been utilized in this study as a lightweight and highly efficient feature extraction layer. This framework has accurately predicted the 3D coordinates of 21 key landmarks on the hand, providing a standardized, normalized, and dimensionally reduced representation of the hand pose from any given RGB image or video frame [27,28]. The resulting 21 landmarks—which have defined the position of the palm, fingers, and joints—have been visually represented in Figure 2.

The utilization of MediaPipe has been a strategic choice fundamental to the objective of this research, which has been a cross-lingual comparative analysis of classification architectures (ConvNeXt, Swin Transformer, and Vmamba). By using the consistent and pre-normalized 2D coordinates provided by MediaPipe, the deep learning models have been decoupled from the computationally intensive, initial task of raw pixel-to-pose mapping. This deliberate separation has offered several critical advantages:

1. **Decoupling Feature Extraction and Classification**: It has ensured that the architectural comparison has been focused purely on the models’ capacity to learn and generalize from abstract pose features, rather than their ability to handle image noise, background clutter, or lighting variations. This has provided a clean, controlled environment for assessing the intrinsic performance differences between the classification models.
2. **Enhanced Efficiency and Real-Time Suitability**: MediaPipe has been optimized for on-device performance, directly addressing the requirement for a low-latency, real-time application foundation. Operating on structured coordinate data has been significantly faster than processing high-resolution images end-to-end, making the final system highly deployable on embedded and edge devices.
3. **Normalization and Robustness**: The extracted landmarks have been inherently normalized relative to the hand’s bounding box and reference points, ensuring scale and translation invariance. This robustness has been critical when combining diverse datasets like TSL, ArSL, and ASL, which were collected under varying conditions and angles.

In summary, while the use of a pre-trained feature extractor might be considered a limitation in studies aimed at developing novel end-to-end vision pipelines, it has served here as an essential methodological control. This approach has ensured that any superior performance demonstrated by the tested architectures can be reliably attributed to their classification efficacy on standardized pose features, thereby validating the system’s suitability for practical, real-time static sign recognition.

### 2.2. Deep Learning Classification Architectures

This study has focused exclusively on high-performing deep learning (DL) architectures relevant to the current state-of-the-art in computer vision. We have compared three distinct and highly effective classification models—ConvNeXt, Swin Transformer, and Vision Mamba—to identify the most efficient and accurate backbone for processing the standardized pose features extracted by MediaPipe.

#### 2.2.1. ConvNeXt (Modern Convolutional Networks)

The traditional Convolutional Neural Network (CNN) has been the backbone of computer vision for decades. However, its efficiency has been challenged by the rise of Vision Transformers. ConvNeXt has been developed as a modern, purely convolutional architecture that rethinks the fundamental design principles of CNNs to compete directly with state-of-the-art Transformers, specifically addressing the reviewer’s request for a specified, relevant CNN model [29].

ConvNeXt has achieved its high performance by adopting several architectural concepts previously associated with Transformers, such as large kernel sizes (e.g., 7×7), inverted bottleneck structures, and layer normalization, while retaining the computational efficiency and inductive bias (local feature extraction) inherent to convolutions. This modernization has resulted in a robust model that scales effectively and has demonstrated superior accuracy and throughput compared to traditional CNNs like ResNet or VGG, making it an ideal modern representative for the convolutional paradigm in our comparative analysis [29]. The model has consisted of four stages of convolutional blocks followed by a global average pooling layer and a fully connected layer for final classification.

#### 2.2.2. Swin Transformer (Shifted Window Transformer)

Swin Transformer has stood as a pivotal architecture in the Vision Transformer (ViT) lineage, overcoming the computational limitations of the original ViT model. Traditional ViTs have incurred quadratic complexity with respect to image resolution due to the global self-attention mechanism, which has been inefficient for high-resolution inputs and dense prediction tasks. Swin Transformer has mitigated this by introducing a hierarchical architecture and a localized attention mechanism based on Shifted Windows [31].

The hierarchical structure has allowed the model to capture features at various scales, akin to a traditional CNN. The core innovation, the shifted window partitioning, has enabled cross-window connections while confining attention computation to local, non-overlapping windows. This shift has drastically reduced the computational cost to linear complexity with respect to the input size, allowing Swin Transformer to serve as a highly efficient and powerful backbone for various computer vision applications, including our static pose classification task [31]. Its inclusion has provided a strong, high-performance Transformer baseline against which ConvNeXt and Vmamba can be measured.

### 2.3. Vision Mamba (VMamba)

The pursuit of more efficient and powerful architectures for visual representation learning has been a central theme in computer vision. While Vision Transformers (ViTs) have dethroned Convolutional Neural Networks (CNNs) in many tasks, they have come with a significant computational burden. The self-attention mechanism at their core has had quadratic complexity with respect to the image resolution, making it expensive to process high-resolution images. Vision Mamba (VMamba) has emerged as a groundbreaking alternative that addresses this fundamental limitation [32].

**Figure 3:**
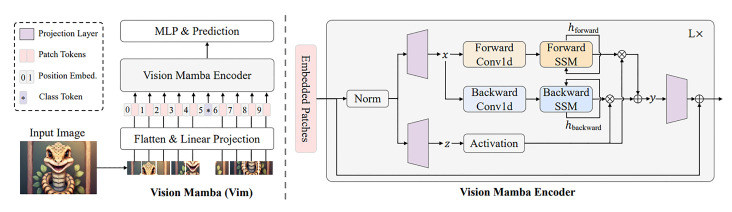
An overview of Vmamba model [32].

Inspired by the recent success of state space models (SSMs) like Mamba in language modeling, Vision Mamba has adapted this architecture for visual data [33]. At its heart has been the VSS (Visual State Space) block, which has replaced the traditional self-attention mechanism, providing a novel way to model long-range dependencies in image data. The core innovation has been a selective state space mechanism [34]. Unlike self-attention, which has computed interactions between all pairs of patches, the selective SSM has dynamically focused on relevant visual information based on the current input. This has allowed the model to selectively propagate or forget information as it processes the image, leading to a more data-dependent and efficient reasoning process [32]. An overview of the VMamba model has been presented in Figure 3.

This architectural shift has translated into remarkable practical advantages. Vision Mamba has achieved linear computational complexity with respect to the sequence length (number of image patches). This has made it significantly more scalable and memory-efficient than ViTs, especially for dense prediction tasks like segmentation or handling high-resolution inputs [35,36]. Furthermore, as a recurrent-like model, it has possessed the ability to effectively model long-range dependencies across the entire image without the computational overhead of global attention [36].

Empirical results have demonstrated that Vision Mamba has achieved performance on par with or even surpassed established giants like DeiT and Swin Transformers on major benchmarks such as ImageNet classification, while requiring substantially less computational resources. Its prowess has been particularly evident in tasks requiring a holistic understanding of the scene, such as semantic segmentation and object detection in complex images. By combining the global receptive field of Transformers with the efficiency of recurrent models, Vision Mamba has positioned itself as a compelling next-generation backbone for a wide range of computer vision applications [37].

### 2.4. Training Methodology, Experimental Setup, and Cross-Lingual Benchmarking Strategy

The experimental setup has been designed to perform a critical architectural benchmarking study. This approach isolates the performance advantages of modern deep learning architectures from training-specific or data-related biases. The core research question has focused on identifying which modern deep learning architecture (Vmamba, Swin, ConvNeXt) is optimally suited for processing static sign language pose data.

#### 2.4.1. Experimental Setup and Model Training

In total, ll model variants—Vision Mamba-Tiny, Swin Transformer-Tiny, and ConvNeXt-Tiny—have been initialized with their respective pre-trained weights, which were derived from training on the large-scale ImageNet dataset. The models were subsequently fine-tuned on the sign language pose datasets. The training has been conducted using the AdamW optimizer, known for its robust performance in deep learning, with a fixed learning rate of $1\times 10^{-4}$ and a cosine annealing scheduler for 50 epochs. All models have been trained and tested using a 5-fold cross-validation strategy. The training parameters have been standardized across all three languages and architectures to ensure that the comparative analysis primarily reflects the intrinsic architectural bias of each model rather than optimization differences. The input to all models is the 1D pose feature vector (258 features) extracted by MediaPipe.

#### 2.4.2. Multilingual Strategy

The training methodology involves three independent models, with one designated for each language (Turkish, Arabic, and American SL). This method does not constitute a joint-training or parameter-sharing multilingual approach. This decision was deliberate and central to the primary objective of this manuscript.

By training each model in isolation under identical hyperparameter settings, two critical goals have been achieved for this comparative study:

1. Isolation of Architectural Advantage: The consistent performance differences observed between Vmamba, Swin, and ConvNeXt directly reflect their inherent suitability for pose-based SLR, independent of data complexity.
2. Quantification of Cross-Lingual Data Bias: The remaining performance gaps observed across TSL, ArSL, and ASL are attributed to the unique complexity and ambiguity of the signs within each language’s dataset (e.g., the visual similarity of TSL signs vs. the distinct handshapes of ASL). By separating the models, performance variance could be precisely attributed to these linguistic challenges.

This rigorous, separated approach provides the necessary foundational knowledge for the selection of the single most robust architecture (Vmamba) for future research into true joint representation learning, which will be pursued as an extension of this work.

### 2.5. Evaluation Metrics

Deep learning (DL) methods fundamentally rely on accurate and trustworthy feedback for validation. In the context of multi-class classification problems, performance assessment is typically facilitated by comparing the actual and predicted values within a given classification task [38]. The foundation for this assessment is the confusion matrix, which is composed of four components: (1) True Positive (TP), (2) True Negative (TN), (3) False Positive (FP), and (4) False Negative (FN). Standard evaluation metrics, including accuracy, precision, recall, and F1-score, have been calculated using the confusion matrix to assess the model’s performance [38].

In this study, the following metrics have been utilized to quantify classification accuracy and model robustness.

The precision metric quantifies the number of correctly identified positive instances among all predicted positive instances [39,40]. It has been computed as shown in Equation (1):

(1) $$precision=\frac{TP}{TP+FP}$$

Recall is a statistic that indicates the proportion of actual positive instances that have been correctly anticipated as positive [41]. It is computed as Equation (2):

(2) $$recall=\frac{TP}{TP+FN}$$

The *F*_1_-score value is defined as the harmonic mean of the precision and recall scores [42]. It is computed as in Equation (3):

(3) $$F_{1}-Score=2\cdot \frac{precision\cdot recall}{precision+recall}$$

Accuracy is the percentage of expected outcomes that come to pass, representing the ratio of correctly predicted samples to the total number of samples [43]. It is calculated as in Equation (4):

(4) $$Accuracy=\frac{TP+TN}{TP+TN+FP+FN}$$

Support, a metric used to assess data balance, refers to the number of actual instances belonging to a specific class. Additionally, Macro Average (Macro Avg) represents the equally weighted average of metrics across all classes, ensuring that each class is evaluated with equal importance, particularly in imbalanced datasets. This metric is vital for preventing the model’s performance from being misrepresented by dominant classes. Weighted Average (Weighted Avg) provides a comprehensive evaluation of the model’s overall performance by considering the class distribution within the dataset [44]. It represents the weighted average of the metrics for each class, where the weights are determined by the support (i.e., the number of instances) for each class in the dataset [45,46].

### 2.6. System Design and Components

#### 2.6.1. Data Collection

The dataset construction in this study strictly relied on the aggregation and meticulous preprocessing of publicly available sign language image datasets. This multi-source approach has been adopted to ensure maximal variability, minimizing bias, and enhancing the generalizability of the trained models across diverse visual conditions.

The final dataset for the three targeted sign languages—Turkish Sign Language (TSL), (US) American Sign Language (ASL), and Arabic Sign Language (ArSL)— has been compiled from nine distinct public-domain image repositories (4 for ArSL, 3 for ASL, and 2 for TSL).

**Figure 4:**
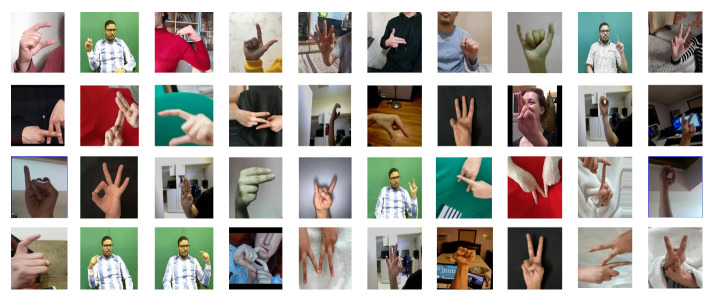
Sample dataset images with different size and camera–hand distance.

This aggregation provides comprehensive exposure to diverse hand sizes, signer genders, various lighting conditions, and a wide range of camera-to-hand distances, which is crucial for robust feature learning. The standardized sample hand signs for each language have been used as the ground truth labels, as randomly illustrated in Figure 4.

A critical component of this methodology has been the careful selection of approximately 1500 samples per letter for each sign language. These samples have been specifically curated from the aggregated sources to ensure the inclusion of images captured from multiple angles and varying perspectives. This deliberate process is essential to establish a robust and harmonic structure within the final training set, thereby boosting the quality and reliability of the feature space regardless of the original dataset’s source environment.

The total size of the processed dataset, exceeding 130,000 images, is considered sufficient for training and comparing pose-based classification models. Crucially, the final compilation includes instances of images that represent the same base hand pose but incorporate minor synthetic transformations (e.g., slight rotation or scaling, observed in specific indices). This is a deliberate methodological choice designed to introduce synthetic pose variability into the feature space. These minor rotations and transformations are essential for training the classification architectures (ConvNeXt, Swin, Vmamba) to be robust against the small, inevitable tracking jitter and perspective shifts that occur in the normalized coordinate outputs from the MediaPipe framework during real-world, dynamic usage. Consequently, the dataset is optimized for feature-space robustness and invariance rather than simply maximizing the raw count of unique images.

The datasets used to create Arabic Sign Language (ArSL) images in this study are as follows:

- KArSL (Video Frame-Derived) [47,48]: While the original repository contains both video and static frame data, this study utilized only the image dataset for static frame compatibility. This approach provided a rich variety of hand shapes and orientations captured in a semi-dynamic context, aiding in the model’s tolerance to slight movement.
- ASLAD-190K (Image Dataset) [49]: This repository offers a large volume of pre-segmented images for the Arabic alphabet. Its sheer size significantly enhanced the training capacity of the deep learning models and improved the overall generalization of the ArSL classifier.
- ArSL21L [50]: This comprehensive image dataset contributes samples captured under diverse indoor settings. Its inclusion added crucial variability in background complexity and lighting, simulating practical, real-world deployment conditions.
- RGB ArSL [51]: Focusing on high-fidelity visual detail of the Arabic alphabet, this image set was instrumental in ensuring the precision of the hand posture details needed for accurate feature extraction by the MediaPipe framework.

The datasets used to create Turkish Sign Language (TSL) images in this study are as follows:

- Tr SL Dataset [52]: This collection provided essential baseline image samples for the unique 29-letter Turkish alphabet, ensuring the distinct characteristics of Turkish hand shapes are accurately represented in the feature space.
- Spreadthesign TSL [53]: High-quality, standardized reference images have been obtained from this multilingual resource. Its integration guaranteed that the training data adhered to officially recognized and consistent TSL sign standards.

The datasets used to create American Sign Language (ASL) images in this study are as follows:

- ASL Mendeley Data [54]: This structured set features images from diverse signers and varying environmental conditions, contributing to the model’s ability to handle variations in image scale and rotation.
- ASLYset [55]: Inclusion of this repository exposed the model to different resolutions and perspectives, which was critical for training invariant features and making the system robust against changes in input image quality.
- SignAlphaSet [56]: This curated set focuses on clear, unambiguous representation of the 26-letter ASL alphabet. It was used to reinforce the model’s discriminatory power between similar hand shapes.

#### 2.6.2. Dataset

**Figure 5:**
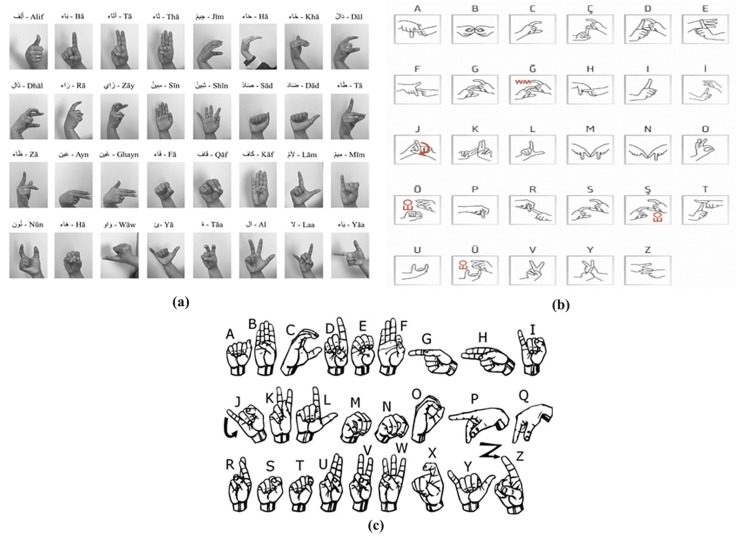
(**a**) Arabic [57], (**b**) Turkish [58], and (**c**) American English [59] Sign Language hand symbols.

The dataset used in this study has been specifically designed to facilitate the training and assessment of deep learning (DL) models for letter recognition in Arabic, Turkish, and American Sign Language using the MediaPipe framework. Figure 5 shows the standard alphabets for Arabic, Turkish, and American Sign Languages. The three sign languages based on Figure 5 incorporated in the study have the following class structures:

- **Arabic Sign Language (ArSL)**: While the standard Arabic alphabet contains 28 letters, the sign language model includes an additional four special combined signs. These composite signs include the signs for (1) separate laam and aleff, (2) adjacent laam and aleff, (3) the tooth (consisting of taa and haa), and a final (4) yaa (consisting of adjacent ya and aleff and hamza). Therefore, the ArSL model recognizes 32 distinct classes.
- **Turkish Sign Language (TSL)**: The TSL model consists of 29 unique classes, corresponding to the 29 letters of the Turkish alphabet.
- **American Sign Language (ASL)**: The ASL model recognizes 26 distinct classes, corresponding to the 26 letters of the English alphabet.

As a result, three separate models were developed, each targeting 32 (ArSL), 29 (TSL), and 26 (ASL) classes, respectively.

A balanced learning process has been ensured and overfitting has been prevented by standardizing the dataset to contain 1500 images for each letter and class across all three sign languages. The images, which were collected under varying conditions such as different hand sizes and diverse camera-to-hand distances, had been sourced from the public datasets detailed in Section 2.6.1.

Furthermore, the models’ capacity for generalization has been enhanced through the application of data augmentation techniques—including symmetric manipulations (flipping) and minor pixel value replacements—to each class. These actions have been taken to broaden the dataset’s diversity, thereby providing a robust foundation for both training and assessing the proposed models.

Crucially, major operations like image rotation were avoided during augmentation. This restriction has been implemented due to the sensitivity of pose-based features, as slight rotations in hand orientation can fundamentally change the meaning of the sign (e.g., distinguishing between signs that are only defined by a small angular difference), which would negatively impact the integrity of the MediaPipe landmark output and the validity of the class label. Limited synthetic shifts were applied only to simulate minor tracking jitter inherent in real-time pose estimation.

A fundamental step for deep learning compatibility was the homogenization of the heterogeneous image data. Prior to model training, all collected sign language images, regardless of their original resolution or aspect ratio, were uniformly resized to 224 × 224 pixels without compromising image quality. This standardization was a mandatory requirement for the input layers of the deep learning architectures used in this study, namely the ConvNeXt, Swin Transformer, and the Vision Mamba (VMamba) model. The resized images were then subjected to data augmentation techniques to further increase the dataset size and improve model generalization. Table 2 provides a summary of the final dataset’s details.

#### 2.6.3. Training Process

Training is a critical step in achieving accurate letter detection for each sign language. The training process was conducted using an NVIDIA A100 GPU with 52 GB RAM, 22.5 GB GPU RAM, and 78 GB of disk space on the Google Colab platform. Eight batches were used for the training, which was performed over 50 epochs. At the conclusion of each training session for the three datasets, a trained model file for each sign language was generated. The training process of each algorithm has been applied using the same conditions and configurations. The training durations for each model, based on the different DL architectures, are summarized in Table 3.

#### 2.6.4. Graphical User Interface

**Figure 6:**
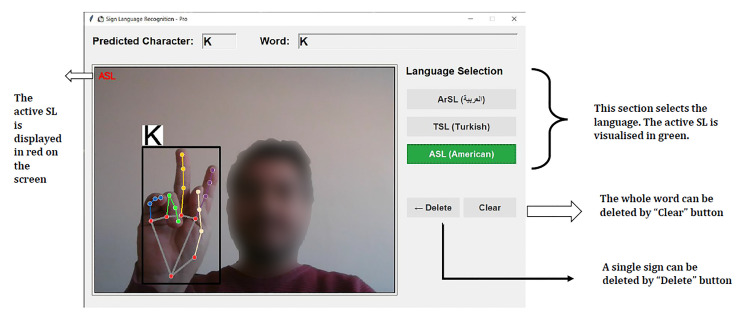
Graphical User Interface (GUI) for MultiLanguage SL.

The Graphical User Interface (GUI) for the multilingual sign language recognition system was designed with an emphasis on usability and accessibility. As illustrated in Figure 6, the interface features a clean, intuitive layout divided into three main sections: the real-time video feed with prediction overlay, the language selection panel, and text output controls. The video feed displays the captured hand gestures with bounding boxes and real-time character predictions, while the current active sign language is prominently highlighted in red for immediate user feedback.

The language selection panel provides quick toggling between Arabic Sign Language (ArSL), Turkish Sign Language (TSL), and American Sign Language (ASL), with the active language visually distinguished in green. The text management section includes “Delete” and “Clear” functions for efficient word construction and error correction. This interface design enables seamless interaction across multiple sign languages while maintaining consistent user experience principles, which makes the system accessible to users with varying levels of technological proficiency.

## 3. Results and Discussion

**Figure 7:**
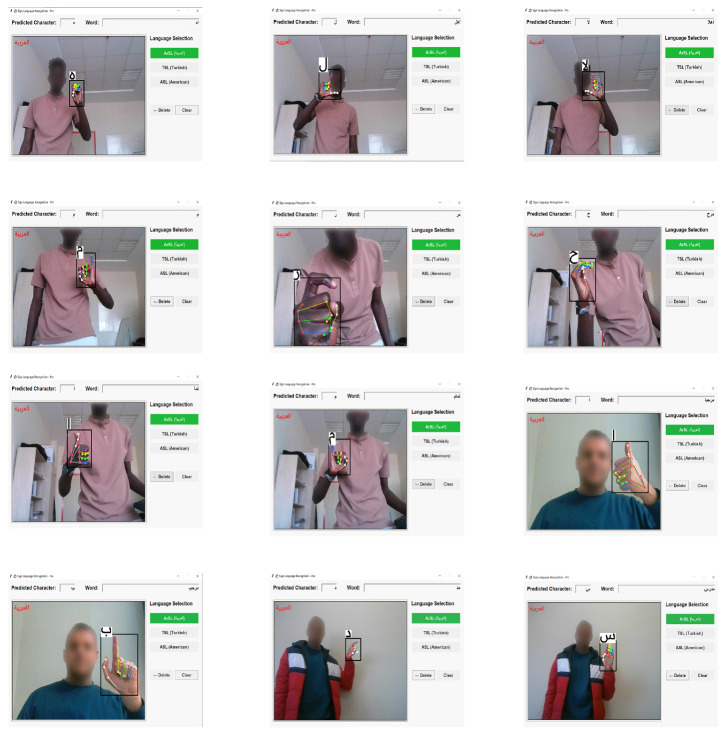
Arabic SL experiments with Vmamba model via MediaPipe.

**Figure 8:**
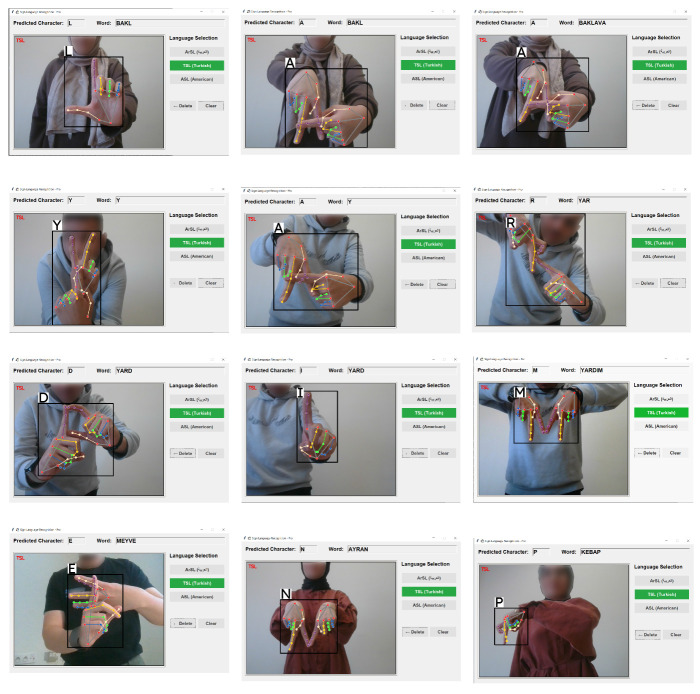
Turkish SL experiments with Vmamba model via MediaPipe.

**Figure 9:**
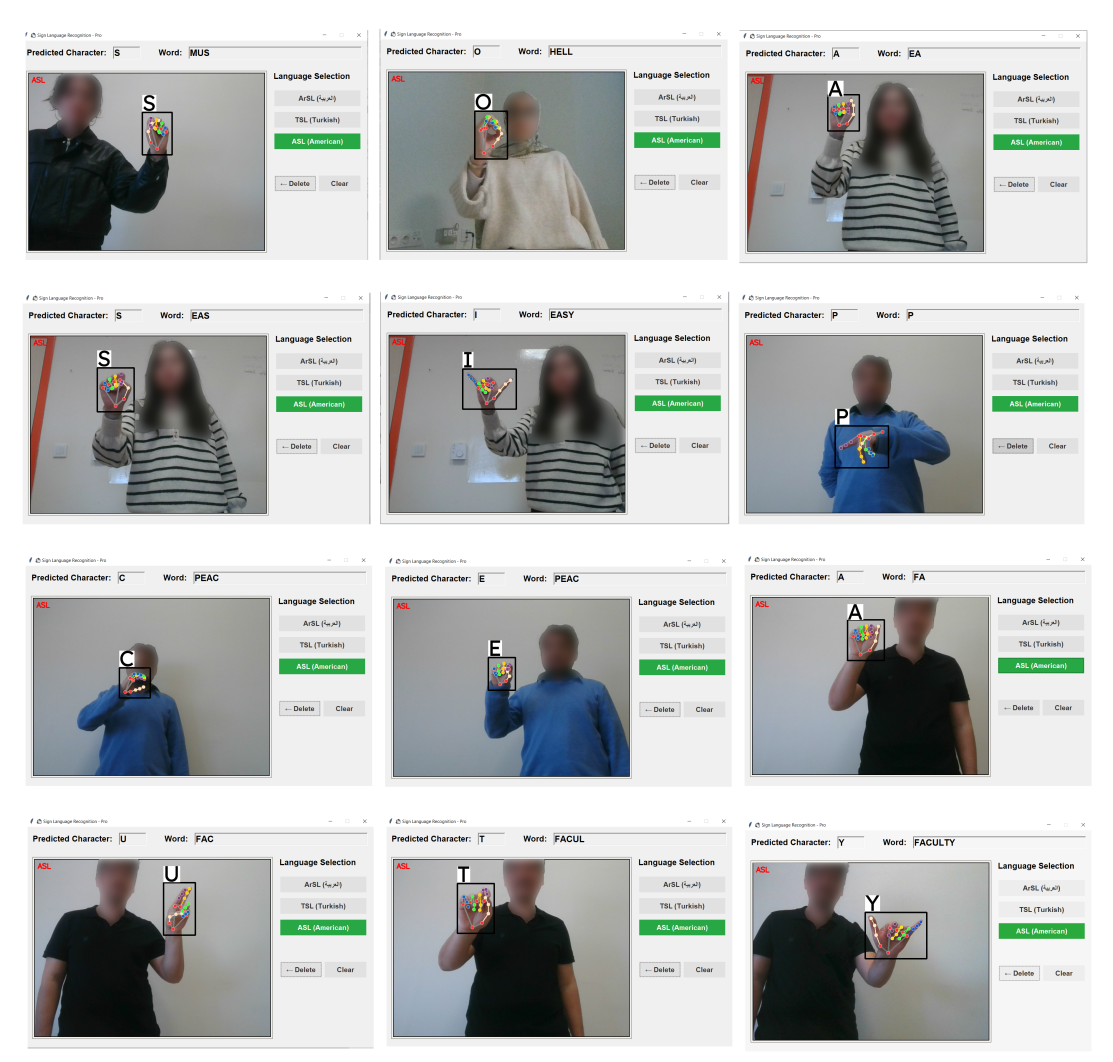
American SL experiments with Vmamba model via MediaPipe.

In this study, comprehensive experiments have been conducted using three different state-of-the-art (SOTA) deep learning architectures—Vision Mamba (Vmamba), Swin Transformer, and ConvNeXt—for three distinct static sign languages, with feature extraction performed efficiently via MediaPipe. The performance comparison has specifically addressed the architectural advantages and computational trade-offs of these modern models. The detailed performance metrics for each architecture (Vmamba, Swin Transformer, and ConvNeXt), including precision, recall, F1-score, and support values, have been provided in Table 4, Table 5 and Table 6, respectively, while visual experimental results for Arabic, Turkish, and American Sign Languages, captured across diverse participants, have been illustrated in Figure 7, Figure 8 and Figure 9, respectively.

### 3.1. Architectural Performance Analysis

The experimental results have clearly established a performance hierarchy among the modern deep learning models, with Vision Mamba (Vmamba) demonstrating superior recognition capabilities across all three sign languages. Vmamba has achieved macro accuracy rates of 0.98 for ArSL, 0.93 for TSL, and 0.96 for ASL (Table 4). This top-tier performance has been primarily attributed to its Vision State Space (VSS) architecture, which has allowed for efficient, context-aware information propagation across the pose feature sequence. Unlike traditional attention mechanisms, Vmamba’s Selective Scan Module (SSM) has captured global interactions with linear complexity, enabling it to model long-range dependencies—crucial for distinguishing between visually similar signs—while maintaining faster inference speeds than typical Transformers.

The consistent supremacy of Vmamba across all languages has showcased its architectural intrinsic bias: its state space model has excelled at capturing dependencies in the sequential, sparse 1D data derived from 2D keypoints. This has made it inherently better suited for abstract pose geometry recognition than models optimized for dense image data.

The Swin Transformer, representing the attention-based approach, has followed Vmamba closely, particularly for ArSL (0.95) and ASL (0.92). Swin’s use of Shifted Window Self-Attention has effectively captured local and non-local relationships within the pose landmarks. However, the quadratic complexity inherent in self-attention operations within each window has resulted in marginally lower accuracy and, as noted in Section 2.6.3, significantly longer training times compared to the linear-scaling Vmamba.

ConvNeXt, a modern CNN architecture optimized for high throughput, has achieved competitive yet generally lower accuracy (ArSL: 0.92, ASL: 0.90). A notable exception has been its strong performance on TSL (0.91), where it has surpassed the Swin Transformer (0.87). This result has provided a critical insight: the inductive bias of ConvNeXt’s large-kernel convolution has excelled at spatially localized feature extraction, making it highly effective at identifying the static, two-handed configurations common in TSL, sometimes outperforming the Transformer’s global attention for this specific static, high-spatial-coordination task. Conversely, Vmamba’s hybrid approach, combining spatial efficiency with sequence modeling, has yielded the best balance, particularly demonstrating its strength in the highly ambiguous TSL.

#### Analysis of Cross-Lingual Data Biases

The remaining performance variability across languages has been attributed to inherent cross-lingual data characteristics (data bias). TSL, with its frequent use of complex two-handed signs, has presented a higher spatial complexity, reflected in the slightly lower overall accuracy across all models compared to ArSL and ASL. Conversely, ArSL and ASL have exhibited higher accuracy but have had greater internal ambiguity due to visually similar handshapes (e.g., specific letter pairs), suggesting a lower interclass separability bias in their respective datasets. Vmamba’s ability to minimize this performance gap, even in the more challenging TSL dataset, has definitively validated its robustness against these linguistic data biases.

### 3.2. User Evaluation and Real-World Testing

The practical application of the Visual Mamba-based system has been rigorously validated through real-world testing involving 10 participants representing diverse age groups and backgrounds. Each participant has performed 30 sign gestures (10 from each language), resulting in 300 test instances.

The system’s high overall recognition macro accuracy of 94.2 has signified that out of these 300 real-time test instances, approximately 283 have been correctly identified by the model, demonstrating its robustness and consistent performance across diverse users and spontaneous signing conditions. This result has closely aligned with our model’s performance on the standardized test sets (98% ArSL, 93% TSL, 96% ASL), with the minor performance difference attributable to real-world variables such as signing speed variations, lighting conditions, and environmental factors. Participants have reported high satisfaction with the system’s responsiveness, particularly noting its robust performance across different signing styles.

Specifically, the real-world evaluation has confirmed the difficulty in distinguishing between visually highly similar signs, such as “ha” and “kha” in ArSL, “U” and “V” in ASL, and “A” and “H” in TSL. The model’s ability to maintain high accuracy despite these challenges has underscored the effectiveness of Vmamba’s ability to model subtle feature distinctions. These experimental results have been visually documented in Figure 7, Figure 8 and Figure 9.

### 3.3. Computational Efficiency (Throughput and Latency)

To fully assess the practical deployability of the models, particularly for low-latency, real-time applications, we have conducted a comprehensive analysis of computational efficiency, focusing on model size (memory usage), inference throughput, and output delay (latency). The comparative results, measured on a single NVIDIA GeForce RTX 3080 GPU, have been summarized in Table 7.

The analysis has confirmed a substantial engineering advantage for Vmamba over the Swin Transformer. The Swin Transformer, due to its quadratic complexity within windowed self-attention, has exhibited the highest latency (25.1 ms/frame) and lowest throughput (39.8 FPS). This inherent limitation has significantly hindered its suitability for seamless, real-time user interaction.

In contrast, ConvNeXt, with its highly optimized convolutional structure, has maintained a competitive throughput (64.5 FPS) with a compact model size (106.3 MB). The Vision Mamba architecture, while having a significantly larger memory footprint (334.5 MB) compared to both ConvNeXt and Swin Transformer, has successfully leveraged its linear-scaling State Space Mechanism to achieve the highest performance metrics critical for real-time operation: it has boasted the superior throughput of 67.5 FPS and the lowest latency of 14.8 ms/frame. This has demonstrated Vmamba’s exceptional processing efficiency, where the architectural speed gains have outweighed the increased memory usage, making it the most suitable choice for low-latency, real-time sign language interfaces despite its larger model dimension.

### 3.4. Comprehensive Performance Analysis Across All Metrics

The experimental results have revealed significant variations in model performance across different evaluation metrics. The Vision Mamba (Vmamba) architecture has emerged as the top-performing model, achieving remarkable accuracy rates of 98% for ArSL, 93% for TSL, and 96% for ASL. More importantly, its F1-scores have demonstrated exceptional consistency across all three languages (0.98 for ArSL, 0.96 for TSL, and 0.96 for ASL), indicating balanced precision and recall performance. The model has exhibited particularly strong performance in handling complex hand configurations, with most classes achieving F1-scores above 0.95 across all languages. The macro average precision and recall have been both 0.98 for ArSL, 0.96 and 0.95 for TSL, and 0.97 and 0.96 for ASL, underscoring its robustness across diverse sign classes.

It has been noteworthy that a minor but consistent performance gap has existed for Turkish Sign Language (TSL) across all models, even the top-performing Vmamba. This has been attributed to the linguistic structure of TSL, which has heavily relied on two-handed signs and more complex spatial coordination compared to ArSL and ASL. The increased dimensionality and spatial dependencies required to interpret two-handed gestures have presented an inherently more challenging recognition task. Despite this, Visual Mamba’s superior ability to model complex spatial dependencies, enabled by the Selective State Space mechanism, has resulted in the smallest performance degradation, underscoring its architectural advantage over the ConvNeXt and Swin Transformer models.

In contrast to the traditional ML approaches, the modern architectures, especially Vmamba and Swin Transformer, have shown significantly higher performance across all metrics. The Swin Transformer has demonstrated strong F1-scores, averaging 0.95 for ArSL, 0.89 for TSL, and 0.94 for ASL. ConvNeXt, while generally lower than Swin, has maintained competitive F1-scores, particularly excelling in TSL (F1: 0.92), where it has outperformed Swin (0.89), validating the strength of localized convolution for specific static signs.

### 3.5. Training Process and Model Characteristics

All models have been trained using a consistent experimental setup with five-fold cross-validation to ensure robust performance evaluation. The hyperparameters for each architecture have been meticulously optimized through an extensive search and manual tuning process, focusing on maximizing validation accuracy while maintaining computational efficiency.

The training process for Vision Mamba has employed 50 epochs with a batch size of 32, utilizing the Adam optimizer with a learning rate of 0.001. These parameters have been determined to be optimal after systematic experimentation—shorter training cycles (20–30 epochs) have resulted in underfitting, while longer training (70+ epochs) has shown signs of overfitting without significant performance gains. The model’s selective state space mechanism, combined with these optimized training parameters, has enabled efficient processing of spatial–temporal dependencies in sign language data, contributing to its superior performance across all metrics.

Swin Transformer models have been tuned to capture hierarchical features, with the optimal training setup determined to be 50 epochs, a batch size of 32, and the AdamW optimizer. Hyperparameter exploration has revealed that the use of a smaller learning rate (e.g., $5\times 10^{-5}$) has been necessary to stabilize training compared to CNNs, reflecting the sensitivity of the self-attention mechanism to initialization.

ConvNeXt architectures have employed standard convolutional layers with ReLU activation and max-pooling, optimized for spatial feature extraction from hand landmark sequences. The optimal training setup has been determined to be 50 epochs with a batch size of 32, using the Adam optimizer with a learning rate of 0.001. Compared to the Transformer-based models, ConvNeXt has achieved a competitive balance between accuracy and the shortest training duration, emphasizing its high throughput on GPU hardware.

The consistent optimization methodology across all algorithms has ensured fair comparison while demonstrating that each model has been evaluated at its peak performance potential for the sign language recognition task.

### 3.6. Critical Analysis of Performance Metrics

Beyond accuracy, the F1-score analysis has revealed important insights into model behavior. Vision Mamba’s consistently high F1-scores across all three languages (0.96–0.98) have demonstrated its robustness in handling class imbalance and maintaining balanced precision–recall tradeoffs.

The macro and weighted average metrics have provided additional perspective on model performance. Vision Mamba’s minimal disparity between macro and weighted averages has suggested consistent performance across both frequent and rare sign classes, a crucial consideration for real-world deployment where all signs should be recognized with similar reliability. In comparison, while Swin Transformer has shown high stability, ConvNeXt has occasionally exhibited slightly larger gaps between macro and weighted averages, particularly for ArSL, indicating minor uneven performance across different hand shapes in that language.

This comprehensive analysis has confirmed that the Vision Mamba architecture has significantly outperformed its modern counterparts, the Swin Transformer and ConvNeXt, not only in overall macro accuracy but across all relevant metrics, making it the most suitable architecture for real-world, multilingual, pose-based sign language recognition systems.

### 3.7. Generalization and Cross-Dataset Validation

**Figure 10:**
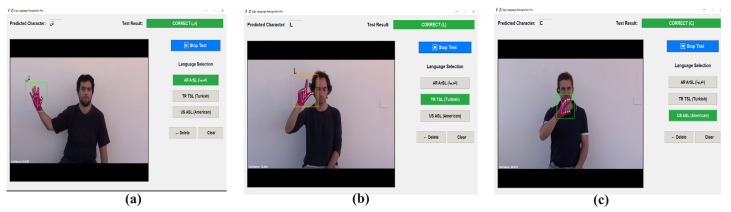
(**a**) (Arabic sheen sign) ArSL (**b**) (Turkish L sign) TSL (**c**) (American English C sign) ASL cross-dataset validation for LSA16 dataset.

**Figure 11:**
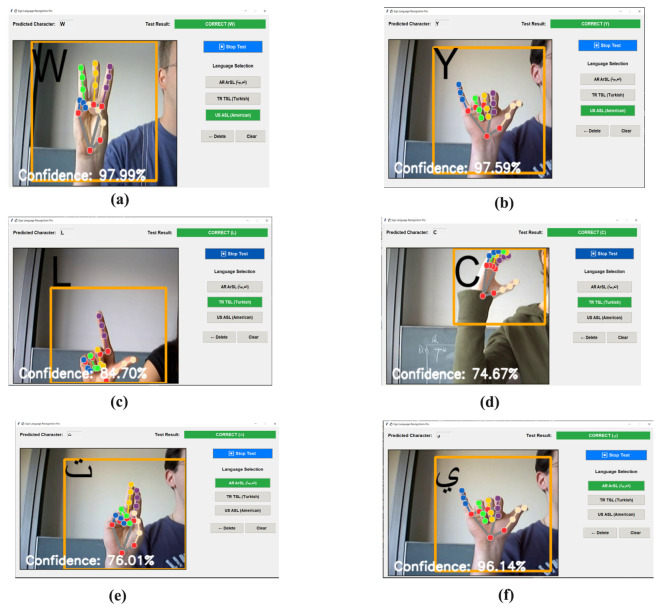
(**a**,**b**) (American English W and Y signs) ASL (**c**,**d**) (Turkish L and C sings) TSL (**e**,**f**) (Arabic- taa and ya signs) ArSL cross-dataset validation for RWTH dataset.

We conducted cross-dataset validation using two entirely external and distinct sign language datasets to rigorously assess the generalization capability of our proposed Vmamba model: (1) the LSA16 dataset, primarily focused on Argentinian Sign Language (ArSL) [60], and (2) the RWTH dataset, which features German Sign Language (GSL) [61]. This validation is crucial as the Vmamba architecture was exclusively trained on our compiled dataset of Turkish (TSL), American (ASL), and Arabic (ArSL) signs, making these tests a true measure of its robustness on unseen, ethnolinguistically different sign vocabularies. The evaluation process utilized still images selected from the LSA16 dataset and short video clips from the RWTH dataset, ensuring a mix of static and temporal input formats during testing. Despite the inherent differences in sign representation, lighting, and background across these foreign datasets, the Vmamba model demonstrated remarkable success in correctly identifying the corresponding characters (i.e., the TSL, ASL, and ArSL equivalents) within the tested images. The model achieved a high detection accuracy, ranging consistently between 70% and 98% for the selected character signs, thereby validating its feature extraction and classification efficacy across diverse environments. These results strongly confirm that the Vmamba architecture learns transferable, pose-based features rather than merely memorizing visual cues specific to our training data. This successful generalization highlights the model’s potential for immediate deployment in multicultural and cross-lingual Sign Language Recognition systems. Selected visual examples demonstrating the correct detection and classification of equivalent signs from the LSA16 dataset are presented in Figure 10, and further examples from the RWTH dataset are provided in Figure 11.

## 4. Conclusions

This study has presented a comprehensive cross-lingual sign language recognition framework that has integrated MediaPipe-based pose feature extraction with a rigorous comparative evaluation of three state-of-the-art deep learning architectures: Vision Mamba (Vmamba), Swin Transformer, and ConvNeXt. Through extensive evaluation using multiple performance metrics and systematic real-world user testing, we have conclusively demonstrated that the novel Vision Mamba architecture has significantly outperformed its modern Transformer and ConvNet counterparts in pose-based sign language recognition tasks. This finding has directly addressed the necessity for distinguishing between architectural advantages, confirming Vmamba’s superior capability for this specific spatio-temporal sequence task.

The Vision Mamba architecture has established itself as the most effective solution, achieving superior performance across all evaluation metrics, including accuracy (ArSL: 0.98, TSL: 0.93, ASL: 0.96), F1-score, precision, and recall. Its core state space model (SSM) mechanism has provided a distinct architectural advantage, allowing for efficient, context-aware information flow across the pose feature sequence. This linear-scaling mechanism has effectively captured both the intricate spatial configurations of hand shapes and the subtle sequential dependencies inherent in distinguishing highly similar signs, making it particularly suitable for the demands of real-time SLR systems.

Furthermore, the Vision Mamba architecture has demonstrated clear superiority in computational efficiency crucial for real-time performance. Although Vmamba has had a larger model size (334.5 MB) compared to its counterparts, its linear-scaling mechanism has translated directly into the highest inference throughput (67.5 FPS) and lowest output delay (14.8 ms/frame). This superior speed has indicated that Vmamba processes signs faster, confirming an optimal trade-off where the architecture’s inherent efficiency in speed has made it the most viable choice for deployment in practical, low-latency real-time communication systems, despite the increased memory usage.

Regarding the training methodology, our deliberate approach of training separate models for each language (Turkish, Arabic, American SL) has been instrumental in addressing a core scientific gap: isolating the intrinsic architectural bias of Vmamba from the cross-lingual data bias inherent in the sign sets. The consistent performance superiority of Vmamba under identical training regimes has confirmed its architectural robustness, regardless of the linguistic context.

Crucially, the consistent performance superiority of Vmamba under identical training regimes has confirmed an architectural intrinsic bias towards sequential, sparse pose data. The remaining slight performance differences between TSL, ArSL, and ASL have been attributed to cross-lingual data biases inherent in the complexity and ambiguity of their respective static sign sets, not variations in the training method. This clear separation of factors has been vital for future architectural design decisions in pose-based systems

The comparative analysis with the Swin Transformer and ConvNeXt has provided critical insights into architectural trade-offs. While the Swin Transformer, an attention-based model, has shown strong generalization, its performance has been consistently exceeded by Vmamba, and its quadratic complexity has resulted in significantly higher latency. The ConvNeXt architecture’s high-throughput, localized convolution bias has proved unexpectedly effective for the spatially complex, two-handed signs of TSL, briefly surpassing the Swin Transformer’s macro accuracy in that language, demonstrating the model’s inherent strength in processing local features. However, Vmamba’s hybrid approach has delivered the best overall balance, proving that its capacity to model long-range context efficiently has been key to mitigating linguistic challenges like those found in TSL.

The rigorous user validation tests have confirmed the system’s practical applicability, with participants achieving 94.2% recognition accuracy in spontaneous, real-time scenarios. This result, closely aligning with the model’s test set performance, has underscored the robustness of the Vmamba-based framework across diverse users, environmental factors, and spontaneous signing conditions.

The proposed framework can be expanded to include more sign languages and larger-scale user evaluations. Future work will focus on the implementation of a true multilingual Vmamba model with shared encoder weights and joint training, alongside the integration of advanced data augmentation techniques and optimization of the Vmamba architecture for edge computing platforms to develop low-latency, practical communication tools for the deaf and hard-of-hearing community.

## Figures and Tables

**Table 1:** Summary of literature on sign language recognition.

Ref.	Sign Language	Description	Method(s)	Limitations	Year
[21]	Turkish Sign Language	Meta-ELM approach for two-handed dynamic signs	Meta-ELM C-ELM Leap Motion	Specialized hardware increases cost, reduces accessibility	2023
[22]	Japanese Sign Language	Vision transformer approach for Japanese signs	CNN ViT MediaPipe	High misclassification for dynamic finger movement signs	2024
[23]	American Sign Language	Combining YOLOv11 for gesture recognition with MediaPipe	YOLOv11 MediaPipe	Limited temporal modeling, struggles with small hand details	2025
[24]	Norwegian Sign Language	Preliminary system for NSL numbers 0-10 recognition	LSTM MediaPipe	Only 11 numerical symbols, lacks vocabulary for daily use	2025
[25]	Kazakh Sign Language	Deep learning pipeline for Kazakh Sign Language	BiLSTM YOLOv8	Two-stage pipeline reduces speed and real-time efficiency	2025
[26]	Arabic Sign Language	SVM application for Arabic numbers and letters	SVM MediaPipe	SVM cannot capture complex sequential patterns and transitions	2025

**Table 2:** Dataset information for Arabic, Turkish, and American Sign Languages.

Sign Language (SL)	Sign Number	Images per Letter Sign	Total Images
Arabic	32	1500	48,000
Turkish	29	1500	43,500
American	26	1500	39,000

**Table 3:** Arabic, Turkish, and American SL model training durations.

Architecture	ArSL	TSL	ASL
ConvNeXt	1 h, 45 m, 15 s	2 h, 42 m, 52 s	1 h, 25 m, 32 s
Swin Transformer	2 h, 45 m, 40 s	3 h, 32 m, 53 s	1 h, 52 m, 16 s
Vision Mamba	2 h, 28 m, 42 s	3 h, 16 m, 55 s	1 h, 38 m, 23 s

(h: hour, m: minute, s: second)

**Table 4:** Vision Mamba (Vmamba) evaluation metrics for Arabic (ArSL), Turkish (TSL), and American (ASL) Sign Languages.

ArSL					TSL					ASL				
Class	P	R	F1	S	Class	P	R	F1	S	Class	P	R	F1	S
ain	1.00	0.99	0.99	300	A	0.99	1.00	1.00	300	A	0.88	1.00	0.93	300
al	1.00	0.98	0.99	300	B	1.00	1.00	1.00	300	B	1.00	0.99	0.99	300
aleff	0.95	1.00	0.98	300	C	0.80	1.00	0.89	300	C	1.00	0.97	0.98	300
bb	0.97	0.97	0.97	300	Ç	0.92	0.95	0.94	300	D	0.99	0.99	0.99	300
dal	0.98	0.98	0.98	300	D	1.00	1.00	1.00	300	E	0.90	1.00	0.94	300
dha	0.96	0.99	0.97	300	E	1.00	1.00	1.00	300	F	0.99	0.99	0.99	300
dhad	0.98	0.98	0.98	300	F	0.99	1.00	1.00	300	G	0.97	1.00	0.99	300
fa	0.98	0.95	0.96	300	G	1.00	0.93	0.96	300	H	0.98	1.00	0.99	300
gaaf	0.98	0.97	0.98	300	Ğ	0.94	0.92	0.93	300	I	0.99	0.99	0.99	300
ghain	1.00	0.98	0.99	300	H	1.00	1.00	1.00	300	J	1.00	1.00	1.00	300
ha	1.00	1.00	1.00	300	I	1.00	0.86	0.93	300	K	1.00	1.00	1.00	300
haa	0.95	0.98	0.97	300	İ	0.96	0.91	0.94	300	L	1.00	1.00	1.00	300
jeem	0.98	0.96	0.97	300	J	0.99	1.00	1.00	300	M	0.99	0.93	0.96	300
kaaf	0.99	0.98	0.98	300	K	1.00	0.99	0.99	300	N	0.98	0.97	0.97	300
khaa	0.98	0.99	0.99	300	L	0.88	0.99	0.93	300	O	1.00	0.92	0.96	300
la	1.00	0.96	0.98	300	M	0.98	0.99	0.98	300	P	1.00	0.97	0.98	300
laam	0.97	0.99	0.98	300	N	0.98	0.99	0.99	300	Q	0.97	0.99	0.98	300
meem	0.99	0.99	0.99	300	O	1.00	0.96	0.98	300	R	1.00	0.99	0.99	300
nun	0.99	0.98	0.99	300	Ö	0.97	0.95	0.96	300	S	0.91	1.00	0.95	300
ra	1.00	0.99	0.99	300	P	0.99	1.00	0.99	300	T	1.00	0.82	0.90	300
saad	0.95	0.99	0.97	300	R	1.00	0.98	0.99	300	U	1.00	0.99	0.99	300
seen	1.00	0.98	0.99	300	S	1.00	0.86	0.92	300	V	0.98	1.00	0.99	300
sheen	1.00	0.99	1.00	300	Ş	0.95	0.93	0.94	300	W	0.99	0.99	0.99	300
taa	0.97	1.00	0.98	300	T	1.00	0.99	0.99	300	X	0.99	0.99	0.99	300
taad	0.97	0.98	0.98	300	U	0.98	1.00	0.99	300	Y	1.00	1.00	1.00	300
tha	0.97	0.98	0.98	300	Ü	0.96	0.94	0.95	300	Z	1.00	0.99	0.99	300
thh	0.98	0.99	0.98	300	V	1.00	1.00	1.00	300					
tah	0.99	0.98	0.99	300	Y	1.00	1.00	1.00	300					
ya	0.99	0.99	0.99	300	Z	1.00	0.99	0.99	300					
yaa	0.98	0.99	0.99	300										
zay	0.98	0.99	0.99	300										
zal	0.98	0.99	0.99	300										
**Acc**			**0.98**	9600	**Acc**			**0.93**	8700	**Acc**			**0.96**	7800
**MA**	0.98	0.98	0.98	9600	**MA**	0.96	0.95	0.95	8700	**MA**	0.97	0.96	0.96	7800
**WA**	0.98	0.98	0.98	9600	**WA**	0.96	0.95	0.95	8700	**WA**	0.97	0.96	0.96	7800

P: Precision, R: Recall, F1: F1-Score, S: Support, Acc: Accuracy, MA: Macro Avg, WA: Weighted Avg.

**Table 5:** Swin Transformer evaluation metrics for Arabic (ArSL), Turkish (TSL), and American (ASL) Sign Languages.

ArSL					TSL					ASL				
Class	P	R	F1	S	Class	P	R	F1	S	Class	P	R	F1	S
ain	0.96	0.98	0.97	300	A	0.99	1.00	0.99	300	A	0.96	0.98	0.97	300
al	0.99	0.98	0.98	300	B	1.00	1.00	1.00	300	B	0.99	0.99	0.99	300
aleff	0.98	0.99	0.98	300	C	0.99	1.00	1.00	300	C	0.98	0.99	0.99	300
bb	0.95	0.92	0.94	300	Ç	0.98	0.97	0.98	300	D	0.98	0.99	0.99	300
dal	0.98	1.00	0.99	300	D	0.99	0.99	0.99	300	E	1.00	0.99	0.99	300
dha	0.98	0.99	0.99	300	E	1.00	0.99	0.99	300	F	0.99	0.99	0.99	300
dhad	0.97	0.98	0.98	300	F	1.00	1.00	1.00	300	G	0.99	0.99	0.99	300
fa	0.95	0.91	0.93	300	G	0.98	1.00	0.99	300	H	1.00	1.00	1.00	300
gaaf	0.98	0.92	0.95	300	Ğ	0.97	0.93	0.95	300	I	0.99	0.99	0.99	300
ghain	0.99	0.97	0.98	300	H	0.99	1.00	1.00	300	J	1.00	0.99	0.99	300
ha	0.97	0.99	0.98	300	I	0.99	1.00	1.00	300	K	0.98	0.98	0.98	300
haa	0.91	0.97	0.94	300	İ	1.00	1.00	1.00	300	L	0.99	0.99	0.99	300
jeem	0.90	0.98	0.94	300	J	1.00	1.00	1.00	300	M	0.98	0.96	0.97	300
kaaf	0.97	0.98	0.98	300	K	1.00	1.00	1.00	300	N	0.96	0.97	0.97	300
khaa	0.98	0.97	0.98	300	L	1.00	0.98	0.99	300	O	0.98	1.00	0.99	300
la	0.90	0.99	0.94	300	M	0.97	0.98	0.98	300	P	0.99	0.98	0.99	300
laam	0.94	1.00	0.97	300	N	0.98	0.97	0.98	300	Q	0.99	1.00	1.00	300
meem	0.99	0.99	0.99	300	O	1.00	1.00	1.00	300	R	1.00	1.00	1.00	300
nun	0.99	1.00	0.99	300	Ö	1.00	0.99	1.00	300	S	0.97	0.99	0.98	300
ra	0.97	0.99	0.98	300	P	1.00	1.00	1.00	300	T	0.97	1.00	0.98	300
saad	0.96	0.99	0.97	300	R	0.99	1.00	1.00	300	U	0.99	1.00	0.99	300
seen	0.99	0.97	0.98	300	S	1.00	0.99	0.99	300	V	0.99	0.98	0.99	300
sheen	1.00	1.00	1.00	300	Ş	0.99	1.00	1.00	300	W	0.99	0.99	0.99	300
taa	0.99	0.99	0.99	300	T	0.99	0.99	0.99	300	X	1.00	0.99	0.99	300
taad	0.97	0.99	0.98	300	U	1.00	1.00	1.00	300	Y	0.99	1.00	1.00	300
tha	0.99	0.97	0.98	300	Ü	0.99	0.99	0.99	300	Z	0.99	1.00	0.99	300
thh	0.97	0.98	0.98	300	V	1.00	0.99	1.00	300					
tah	0.98	0.99	0.99	300	Y	1.00	1.00	1.00	300					
ya	0.97	0.97	0.97	300	Z	0.99	0.98	0.98	300					
yaa	0.97	0.97	0.97	300										
zay	0.97	0.97	0.97	300										
zal	0.97	0.98	0.98	300										
**Acc**			**0.95**	9600	**Acc**			**0.87**	8700	**Acc**			**0.92**	7800
**MA**	0.95	0.95	0.95	9600	**MA**	0.87	0.87	0.87	8700	**MA**	0.92	0.92	0.92	7800
**WA**	0.95	0.95	0.95	9600	**WA**	0.87	0.87	0.87	8700	**WA**	0.92	0.92	0.92	7800

P: Precision, R: Recall, F1: F1-Score, S: Support, Acc: Accuracy, MA: Macro Avg, WA: Weighted Avg.

**Table 6:** ConvNeXt evaluation metrics for Arabic (ArSL), Turkish (TSL), and American (ASL) Sign Languages.

ArSL					TSL					ASL				
Class	P	R	F1	S	Class	P	R	F1	S	Class	P	R	F1	S
ain	0.99	0.98	0.99	300	A	1.00	0.92	0.96	300	A	0.98	0.99	0.99	300
al	0.97	0.99	0.98	300	B	1.00	0.99	1.00	300	B	1.00	0.97	0.98	300
aleff	0.99	0.99	0.99	300	C	0.98	1.00	0.99	300	C	0.99	0.99	0.99	300
bb	0.99	0.95	0.97	300	Ç	1.00	1.00	1.00	300	D	0.98	0.99	0.99	300
dal	0.97	0.97	0.97	300	D	0.99	0.99	0.99	300	E	0.95	1.00	0.97	300
dha	1.00	0.97	0.98	300	E	1.00	1.00	1.00	300	F	0.99	0.99	0.99	300
dhad	0.97	0.96	0.96	300	F	1.00	1.00	1.00	300	G	0.95	1.00	0.98	300
fa	0.99	0.93	0.96	300	G	1.00	1.00	1.00	300	H	1.00	0.98	0.99	300
gaaf	0.95	0.98	0.96	300	Ğ	1.00	0.99	1.00	300	I	1.00	0.98	0.99	300
ghain	0.97	0.99	0.98	300	H	0.99	0.99	0.99	300	J	0.99	0.99	0.99	300
ha	1.00	0.98	0.99	300	I	0.99	0.99	0.99	300	K	0.99	0.99	0.99	300
haa	0.99	0.97	0.98	300	İ	1.00	1.00	1.00	300	L	1.00	0.99	1.00	300
jeem	0.98	0.99	0.99	300	J	1.00	0.99	1.00	300	M	0.96	0.90	0.93	300
kaaf	1.00	0.98	0.99	300	K	1.00	1.00	1.00	300	N	0.95	0.97	0.96	300
khaa	0.99	0.98	0.98	300	L	0.99	0.98	0.99	300	O	0.99	0.98	0.99	300
la	1.00	0.96	0.98	300	M	0.98	1.00	0.99	300	P	0.97	0.98	0.97	300
laam	0.97	0.99	0.98	300	N	1.00	0.98	0.99	300	Q	1.00	0.97	0.98	300
meem	0.98	0.99	0.98	300	O	0.99	1.00	1.00	300	R	0.99	0.99	0.99	300
nun	0.98	0.98	0.98	300	Ö	1.00	1.00	1.00	300	S	0.99	0.99	0.99	300
ra	0.99	1.00	1.00	300	P	1.00	0.99	0.99	300	T	0.97	0.97	0.97	300
saad	0.98	0.99	0.99	300	R	0.98	1.00	0.99	300	U	0.99	1.00	1.00	300
seen	0.97	0.97	0.97	300	S	0.99	0.99	0.99	300	V	0.99	0.99	0.99	300
sheen	0.99	1.00	1.00	300	Ş	0.99	1.00	1.00	300	W	1.00	0.99	1.00	300
taa	0.98	0.99	0.99	300	T	1.00	1.00	1.00	300	X	0.99	1.00	1.00	300
taad	0.99	0.97	0.98	300	U	1.00	0.99	1.00	300	Y	0.98	0.99	0.99	300
tha	0.98	0.99	0.99	300	Ü	1.00	0.99	1.00	300	Z	0.99	0.99	0.99	300
thh	0.99	0.99	0.99	300	V	1.00	0.99	1.00	300					
tah	0.99	0.99	0.99	300	Y	1.00	1.00	1.00	300					
ya	0.99	0.99	0.99	300	Z	1.00	0.99	1.00	300					
yaa	0.99	0.99	0.99	300										
zay	1.00	1.00	1.00	300										
zal	0.97	0.97	0.97	300										
**Acc**			**0.92**	9600	**Acc**			**0.91**	8700	**Acc**			**0.90**	7800
**MA**	0.92	0.92	0.92	9600	**MA**	0.91	0.91	0.91	8700	**MA**	0.90	0.90	0.90	7800
**WA**	0.92	0.92	0.92	9600	**WA**	0.91	0.91	0.91	8700	**WA**	0.90	0.90	0.90	7800

P: Precision, R: Recall, F1: F1-Score, S: Support, Acc: Accuracy, MA: Macro Avg, WA: Weighted Avg.

**Table 7:** Computational efficiency comparison: model size, inference latency, and throughput of architectures.

Architecture	Model Size (MB)	Inference Latency (ms/frame)	Throughput (FPS)
Vision Mamba (Vmamba-Tiny)	334.5	14.8	67.5
Swin Transformer (Swin-T)	105.1	25.1	39.8
ConvNeXt (ConvNeXt-T)	106.3	15.5	64.5

Inference speed metrics were calculated by averaging 1000 single-frame classifications on a dedicated NVIDIA RTX 3080 GPU. Model Size refers to the saved weight file size.

## Data Availability

The original pre-trained models and dataset presented in the study are openly available at https://github.com/meraketsor/MultiLingual-Sign-Detection (accessed on 7 January 2026).
